# Results from a rapid national assessment of services for the prevention of mother-to-child transmission of HIV in Côte d'Ivoire

**DOI:** 10.7448/IAS.19.5.20838

**Published:** 2016-07-20

**Authors:** S Adam Granato, Stephen Gloyd, Julia Robinson, Serge A Dali, Irma Ahoba, David Aka, Seydou Kouyaté, Doroux A Billy, Samuel Kalibala, Ahoua Koné

**Affiliations:** 1 Health Alliance International, Seattle, WA, USA; 2 Department of Global Health, University of Washington, Seattle, WA, USA; 3 School of Social Work, University of Washington, Seattle, WA, USA; 4 Insitut National de la Santé Publique, Abidjan, Côte d'Ivoire; 5 Programme National de Lutte contre le SIDA, Ministère de la Sante et de l'Hygiène Publique, Abidjan, Côte d'Ivoire; 6 Population Council, Washington, DC, USA

**Keywords:** PMTCT Cascade, prevention of mother-to-child transmission of HIV, health systems factors, Côte d'Ivoire, health workforce, patient retention

## Abstract

**Introduction:**

Loss-to-follow-up (LTFU) in the prevention of mother-to-child HIV transmission (PMTCT) programmes can occur at multiple stages of antenatal and follow-up care. This paper presents findings from a national assessment aimed at identifying major bottlenecks in Côte d'Ivoire's PMTCT cascade, and to distinguish characteristics of high- and low-performing health facilities.

**Methods:**

This cross-sectional study, based on a nationally representative sample of 30 health facilities in Côte d'Ivoire used multiple data sources (registries, patient charts, patient booklets, interviews) to determine the magnitude of LTFU in PMTCT services. A composite measure of retention – based on child prophylaxis, maternal treatment and infant testing – was used to identify high- and low-performing sites and determine significant differences using Student's *t*-tests.

**Results:**

Among 1,741 pregnant women newly recorded as HIV-positive between June 2011 and May 2012, 43% had a CD4 count taken, 77% received appropriate prophylaxis and 70% received prophylaxis intended for their infant. During that time, 1,054 first infant HIV tests were recorded. A conservative rate of adherence to antiretroviral therapy was estimated at 50% (*n=*219 patient charts). Significant differences between high- and low-performing sites included: duration of time elapsed between HIV testing and CD4 results (29.5 versus 56.3 days, *p=*0.001); and density (number per 100 first antenatal care visits) of full-time physicians (6.7 versus 1.7, *p=*0.04), laboratory technicians (2.3 versus 0.7, *p=*0.046), staff trained in PMTCT (10.7 versus 4.7, *p=*0.01), and staff performing patient follow-up activities (7.9 versus 2.5, *p=*0.02). Key informants highlighted staff presence and training, the availability of medical supplies and equipment (i.e., on-site CD4 machine), and the adequacy of infrastructure (i.e., space and ventilation) as perceived key factors positively and negatively impacting retention in care.

**Conclusions:**

Patient LTFU occurred throughout the PMTCT cascade from maternal to infant testing, with retention scores ranging from 0.10 to 0.83. Sites that scored higher had more dedicated and trained frontline health workers, and emphasised patient follow-up through outreach and the reduction of delays in care. Strategies to improve patient retention and decrease transmission should emphasise patient tracking systems that utilise critical human resources to both improve data quality and increase direct patient follow-up.

## Introduction

In much of Africa, programmes for the prevention of mother-to-child transmission of HIV (PMTCT) have performed less than optimally [1–4]. Health systems factors cause bottlenecks that impede patient flow through health care services, contribute to inconsistent data quality, and cause patient loss-to-follow-up (LTFU) in various stages of the PMTCT cascade [5,6]. Understanding how pregnant women navigate PMTCT health services and the role of health system factors in disrupting (or facilitating) patient flow helps identify key obstacles to health outcomes and should inform PMTCT programme implementation [7,8]. This approach, at the national scale, is especially pertinent, given that all 21 African priority countries of the Interagency Task Team for PMTCT have adopted one of the World Health Organization's (WHO's) PMTCT Option B/B+ strategies, which include the universal treatment of all HIV-positive pregnant women with combination antiretroviral therapy (cART) [9].

Côte d'Ivoire's *Ministère de la Santé et de l'Hygiène Publique* (MSHP), the Ministry of Health and Public Hygiene, introduced its first nationwide PMTCT programme in 2007, with the goal of lowering the national MTCT rate to 3% by 2015. Despite nationwide decreases in both HIV prevalence and new infections from 2005 to 2013 [10], UNAIDS and the Côte d'Ivoire National AIDS Council estimated that the MTCT rate remained as high as 25% in 2012 [11]. In response, the Côte d'Ivoire MSHP launched the 2012–2016 National PMTCT Scale-up Plan [12], which outlined a transition of national PMTCT treatment standards from WHO's Option A regimen to the Option B regimen [13].

To help inform the rollout of Option B and better understand the challenges of the existing national programme, the MSHP supported a nationwide operations research project to assess PMTCT programme effectiveness. The primary objectives of this project included:

1. Identify factors associated with bottlenecks in Côte d'Ivoire's PMTCT services.
2. Propose test interventions to reduce LTFU and improve PMTCT outcomes under Option B.
3. Help policy makers select and test such interventions during the nationwide rollout of Option B.

The data presented in this paper highlight the major findings from the rapid nationwide assessment to identify major bottlenecks in Côte d'Ivoire's PMTCT cascade under Option A, and to distinguish characteristics of high- and low-performing health facilities. The assessment was carried out with the support of HIVCore, a United States Agency for International Development (USAID)-funded project under the United States President's Emergency Plan for AIDS Relief (PEPFAR), and was implemented in collaboration with Health Alliance International and the University of Washington.

## Methods

Drawing from the 2011 MSHP national PMTCT database of 734 health facilities (public and private) providing PMTCT services in Côte d'Ivoire, 30 sites were selected from among the 320 sites that reported at least 10 HIV-positive pregnant women in 2011. Sites were selected randomly using probability proportional to size sampling, based on the number of women reported HIV-positive in antenatal care (ANC). Seven two-person study teams collected quantitative and qualitative data over the course of two days at each facility in March 2013. Among the 30 randomly selected study sites, 12 (40%) were located in the metropolitan area of Abidjan, 6 (20%) were located in other major urban centres in the country, and the remaining 12 (40%) were located in peri-urban or rural areas distributed throughout the country. At each site, local health facility staff, familiar with on-site registries and patient flow, facilitated data collection.

### Quantitative data

Quantitative data from the following sources were abstracted at each site and recorded in a standardised study tool:

1. On-site health facility registries.
2. Individual patient charts of HIV-positive women.
3. Mother-baby booklets (*carnets*), which are issued to all pregnant women at their first ANC visit (ANC1) to record pregnancy and childhood care.

Registry data were collected from all available registries with relevant data for the period of June 2011–May 2012. This time period ensured that all patient data included in the study pertained to mothers who received care under Option A and had given birth by the date of data collection. Patient chart data were abstracted at each facility from up to 20 patient charts belonging to HIV-positive women tested in PMTCT care services prior to June 2012. Charts were selected systematically in reverse chronological order. Carnet data were abstracted from a convenience sample of up to 20 women who were attending infant immunisation services at the study site on the day of data collection. Project team members processed data collection forms to identify and correct inconsistencies. Table 1 lists illustrative quantitative indicators collected and sample sizes for each data source.

**Table 1:** Data sources and illustrative indicators collected

**Registries**	**Sample:** Monthly data from June 2011–May 2012	**Total number of:** Pregnant women who tested HIV-positive in antenatal care. HIV-positive pregnant women who had a CD4 count taken. HIV-positive pregnant women found eligible for cART. HIV-positive pregnant women newly initiated on cART. HIV-positive pregnant women who received antiretroviral drugs (ARVs) as prophylaxis intended for herself. HIV-positive pregnant women who received ARVs as prophylaxis intended for her infant. First HIV tests administered to HIV-exposed infants
**Patient charts**	**Sample:** Up to 20 charts per facility of women enrolled in PMTCT care in May 2012 or earlier	**Date of:** HIV test Blood draw for initial CD4 count CD4 count results returned to the patient Establishment of eligibility for lifetime cART Initiation of cART Last recorded visit to the health facility
**Carnets**	**Sample:** Up to 20 carnets per facility, from a convenience sample of women seeking newborn immunisations on day of data collection	Type of facility that issued the *carnet* Proof of administration of HIV test (y/n)

### Quantitative analysis

For each facility, a composite outcome measure of PMTCT performance (PMTCT score) was calculated using registry data for 29 of the 30 facilities included in the analysis (see discussion of site #30 below). The PMTCT score was defined as the mean of the following three measures of retention identified by key stakeholders:

1. Proportion of HIV-positive pregnant women who received antiretroviral drug (ARV) prophylaxis intended for her infant.
2. Proportion of HIV-positive pregnant women who initiated cart.
3. Proportion of HIV-positive mothers whose infant had an HIV test performed within one year of birth.

Site characteristics from the 10 facilities with the highest PMTCT scores (High Performers) were compared against the 10 facilities with the lowest PMTCT scores (Low Performers) using a two-sample Student's T-test to identify significant differences (*p*<0.05) between the means of high and low performers. Site characteristics (see Table 2) included quantitative and qualitative measures related to size, location, processes of care and workforce. Workforce characteristics included staff density by cadre, adjusting for patient load at each facility (average ANC1 visits per month) and staff training.

**Table 2:** Site characteristics with data source compared between low- and high-performance group by category

Category	Data source	Characteristic
Size	Facility records	Catchment area population
	ANC registry	Total ANC1 visits
Location	Based on location	Abidjan/non-Abidjan Urban/rural
Processes of care	Patient charts	Average number of days elapsed, according to patient charts, between HIV test and the return of CD4 results to the patient
	Key informant interviews	Reported number of internal displacements per typical patient Reported number of external displacements per typical patient Reported timing of patient enrolment (via patient chart) Reported average number of days between ANC1 reception and establishment of cART eligibility per patient
Workforce distribution	Key informant interviews and ANC registry	Density of full-time health workers (per 100 ANC1 visits) by cadre
Workforce training	Key informant interviews and ANC registry	Density of staff trained in PMTCT or in cART Density of staff who initiate prophylaxis and/or cART Density of staff who engage in ART follow-up

For all site characteristics found to differ substantially between high- and low-performing sites (those with a t-test that resulted in a *p*-value less than 0.10), we used univariate and multivariate logistic regression models with robust standard errors, to determine how those site characteristics were associated with performance, adjusting for available confounders. We determined urban or rural location, catchment and the average number of ANC1 patients per month to be *a priori* potential confounders based on their plausible association with site performance and with each characteristic of interest. These potential confounders were maintained in the model if their inclusion resulted in a change in the effect estimate of approximately 10%. All analyses were performed in Stata 13 IC.

Patient charts were analysed to determine the number of days between the date of the HIV test, CD4 count draw, the return of CD4 results to patient, and the delivery of ARVs. Suspected outliers were eliminated from the analysis using the interquartile range rule for outliers (1.5×IQR). A proxy rate of cART adherence among women on lifelong treatment was calculated as the proportion of charts with a recorded date of patient contact with the health facility within 30 and 90 days of the date of data collection. These cut-offs represent the standard (30 days) and maximum (90 days) number of days of ARVs systematically prescribed in PMTCT care. Carnets do not include an official HIV testing indicator; however, the health facility staff helped identify recognisable codes handwritten into the margins of collected carnets that indicated history of HIV testing.

### Qualitative data

**Figure 1:**
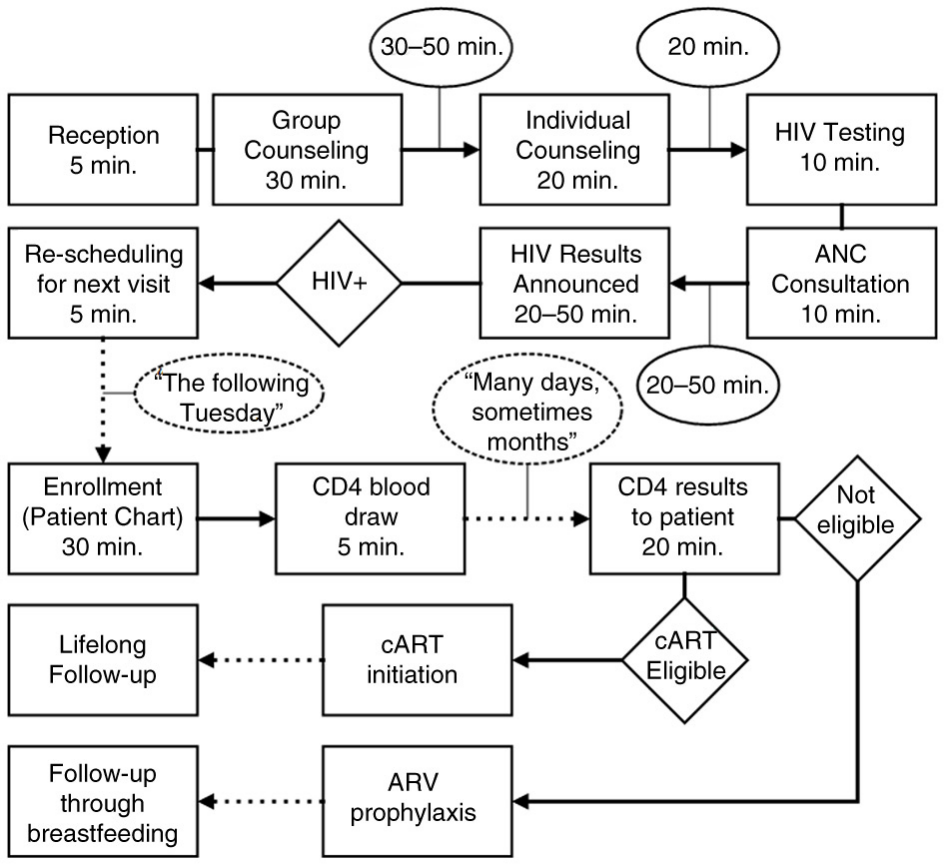
Example of patient flow map. Flow maps were created at each site to chart patient flow through PMTCT services (rectangles), and identify wait times, and internal (solid arrow) and external (dashed arrow) displacements.

Qualitative data were collected at all 30 sites to describe characteristics of the PMTCT cascade and obtain health worker perspectives on perceived facilitators and barriers to the successful completion of PMTCT services. Data collectors conducted semi-structured interviews with one key informant at each site – typically physicians responsible for HIV activities – chosen based on their knowledge of on-site PMTCT services. Key informants were asked to describe each step of PMTCT services and create a patient flow map diagramming the number of internal and external displacements required by patients as they navigate PMTCT services at the facility as shown in Figure 1. An internal displacement was defined as any time a patient must move from one room to another within the same visit, while external displacement indicated any time a patient must return to the site for a follow-up visit. Data collectors took written notes and recorded on-site observations using a standardised study tool.

### Qualitative analysis

Qualitative data were analysed and compared between sites to assess patient flow within PMTCT services, displacements and delays in PMTCT services, and perceived facilitators and barriers to the successful completion of the PMTCT cascade from ANC1 through infant testing. Informants reported barriers and facilitators separately at each stage of patient flow. Two study team members independently extrapolated recurring themes from interview notes to describe the PMTCT cascade, measure delays and identify system facilitators and barriers to service delivery.

### Ethical review

Study procedures, including informed consent, were approved by the National Research Ethics Review Committee of Côte d'Ivoire and the Institutional Review Board (IRB) of Population Council. The University of Washington IRB and the Health Alliance International Ethical Review Committee determined the study to be non-research.

## Results

Data collectors noted a total of 18 different official registries, reports and other unofficial data sources related to PMTCT across the 30-site sample. The study team found substantial variation in the availability and completeness of PMTCT-related indicators in on-site registries, and frequent inconsistencies in the data when compared with the same data aggregated and reported at the national level. Only six sites had no missing data in the 12-month study period. One rural site was excluded from the registry and patient chart-based analyses due to the interruption of PMTCT services during the study period. Further discussion of the availability, quality, and use of these and general on-site data has been discussed elsewhere [14–16].

### Overall LTFU in the PMTCT cascade

At the 29 sites with registry data during the study period, 38,347 women were registered as having attended ANC1, and 42,162 HIV tests were recorded (110% of ANC1 visits). These may have included tests on non-pregnant women and tests conducted on women attending subsequent ANC visits. Of the registered tests, 1741 (4%) were noted positive. Fifteen sites collected additional information on the number of HIV tests administered only during ANC1. Among 19,173 women who attended an ANC1 at these 15 sites, 17,958 (94%) were reported as having received an HIV test during ANC1, of which 889 (5%) tested positive. At all 30 sites, 590 carnets were available and examined. Of these, 489 (83%) carnets had proof of HIV testing during the mother's most recent pregnancy, and 37 (6%) indicated a positive HIV result. The rate of recorded HIV testing in carnets varied by site of first ANC visit; from 87% (*n=*511) in carnets belonging to women who attended ANC at a public MSHP site to 59% (*n=*34) and 55% (*n=*17) in carnets of women who attended ANC1 at non-governmental health facilities and private clinics, respectively.

**Figure 2:**
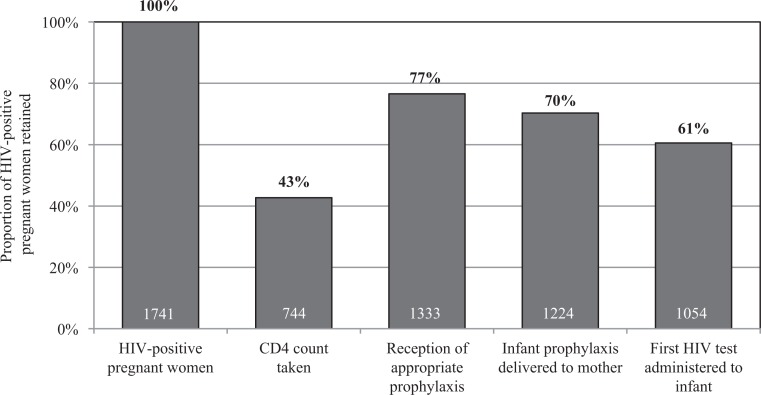
Numbers and percentages of recorded HIV-positive patients reported retained at each step of the PMTCT cascade at 29 health facilities in Côte d'Ivoire.

Figure 2 shows the PMTCT cascade, based on registry-data alone, from the 12-month study period. During this time, 1741 HIV-positive pregnant women were newly diagnosed and registered at 29 sites. Of these, 744 (43%) were reported as having obtained a CD4 count and received their results, of which 209 were registered as eligible for cART (CD4<350). During the same time period, 1,333 (77%) HIV-positive pregnant women received ARVs to reduce MTCT, of which 284 were prescribed lifelong cART. The registries recorded 1,224 (70%) mothers who received ARVs for their infants and 1,054 (61%) who returned to have their infant tested for HIV. Five sites did not report performing any HIV tests on exposed infants during the reporting period.

The receipt of appropriate prophylaxis includes both the delivery of ARV prophylaxis and initiation of cart. To estimate the rates of adherence following cART initiation, a total of 330 patient charts of HIV-positive pregnant women enrolled in PMTCT services were abstracted from the 29 sites. Of the selected charts, 219 belonged to women on lifelong cART and included the date of the last contact with the patient. Only 109 (50%) and 61 (28%) of the 219 charts contained evidence of a visit within 90 or 30 days, respectively.

### Factors associated with loss to follow-up in the PMTCT cascade

The 10 health facilities in the high-performance group had a mean PMTCT score of 0.65 (range: 0.54–0.83) with a mean PMTCT score among low performers of 0.23 (range: 0.10–0.33).

#### Size and location

Even though the average catchment area among high performers was twice the size of the average catchment area among low performers, the average number of monthly ANC1 visits was similar between the two groups. Nevertheless, as seen in Table 3, neither size nor location was found to be significantly different between the two groups.

**Table 3:** Comparison of site characteristics between high- and low-performance groups: size and location

Site characteristic	High performers (mean)	Low performers (mean)	*p*
Catchment area	113,853	56,874	0.22
ANC1 attendance (per month)	108.5	101.4	0.79
Ratio – Abidjan:non-Abidjan	3:7	3:7	1.00
Ratio – Urban: Rural	3:7	4:6	0.66

#### Processes of care

Table 4 summarises site characteristics related to processes of care among high- and low-performing sites. In high-performing sites, the mean number of days between HIV testing and the return of CD4 results to the patient, as reported in patient charts, was nearly half the average time reported at low-performing sites. Similarly, key informant estimates of the time that elapsed from ANC1 to CD4 results, although less overall, were also significantly different between high and low performers. The percentage of sites that followed the MSHP recommendation to open a new PMTCT patient chart immediately following a positive HIV test result was higher among the high-performers; however, the difference was not significant (44% vs. 20%, *p=*0.27).

**Table 4:** Comparison of site characteristics between high- and low-performance groups: processes of care

Site characteristic	High performers (mean)	Low performers (mean)	*p*
Days elapsed: HIV test→CD4 results (recorded in patient charts)	29.5 (22.0, 36.9)	56.3 (40.6, 72.0)	0.001
Days elapsed: ANC1→CD4 results (reported by staff)	5.1 (3.3, 6.9)	11.4 (8.3, 14.6)	0.0009
Patient enrolment time (pre/post CD4)	0.2	0.44	0.27
Internal displacements (typical patient)	5.9	5.5	0.64
External displacements (typical patient)	2.6	2.7	0.66

Confidence intervals (95%) are reported in brackets for characteristics with *p*<0.1.

#### Workforce distribution

Table 5 lists adjusted workforce distribution characteristics (staff density) compared between high- and low-performing sites. Across all technical workforce cadres tested, high-performing sites had more personnel, on average, than low-performing sites. The numbers of health educators and community counsellors were higher on average at low-performing sites. After adjusting for patient load, the density of two health care cadres stood out as significantly different between high- and low-performing sites. These cadres were full-time physicians and laboratory technicians. The magnitude of the difference between the density of nurses at high- and low-performing sites was also noteworthy, although not significant.

**Table 5:** Comparison of site characteristics between high- and low-performance groups: workforce distribution

Site characteristic	High performers (mean)	Low performers (mean)	*p*
Full-time physicians per 100 ANC1 patients	6.5 (0.02, 0.11)	1.8 (0.01, 0.02)	0.04
Laboratory technicians per 100 ANC1 patients	1.9 (0.9, 3.0)	0.7 (−0.2, 1.5)	0.046
Nurses per 100 ANC1 patients	6.1 (1.1, 11.1)	1.9 (0.8, 3.0)	0.09
Midwives	5.7	3.3	0.38
per 100 ANC1 patients			
Nurses aids	4.2	3.4	0.65
per 100 ANC1 patients			
Pharmacy staff	1.3	0.7	0.24
per 100 ANC1 patients			
Health educators	0.2	0.4	0.62
per 100 ANC1 patients			
Community counsellors per 100 ANC1 patients	2.7	6.1	0.50

Data are adjusted for patient load based on the mean number of ANC1 visits per month at each health facility. Confidence intervals (95%) are reported in brackets for characteristics with *p*<0.1.

#### Workforce training

As shown in Table 6, on average, more staff had received training or performed specific follow-up tasks in high-performing sites than in low-performing sites. PMTCT training and staff engagement in patient follow-up activities stood out as characteristics differing significantly between high- and low-performing sites.

**Table 6:** Comparison of site characteristics between high- and low-performance groups: workforce training

Site characteristic	High performers (mean)	Low performers (mean)	*p*
Staff trained in PMTCT per 100 ANC1 patients	10.7 (6.3, 15.1)	4.7 (2.6, 6.7)	0.01
Staff Trained in cART per 100 ANC1 patients	5.8	2.0	0.16
Staff who Initiate cART per 100 ANC1 patients	5.2	2.1	0.18
Staff who conduct follow up per 100 ANC1 patients	7.9 (3.4, 12.4)	2.5 (0.5, 4.4)	0.02
Staff who initiate prophylaxis per 100 ANC1 patients	6.8	4.2	0.22

Data are adjusted for patient load based on the mean number of ANC1 visits per month at each health facility. Confidence intervals (95%) are reported in brackets for characteristics with *p*<0.1.

The inclusion of potential confounders in multivariate logistic regression models did not alter the overall significance of the results observed using the *t*-test for any of the site characteristics evaluated. Although some of the estimated adjusted odds ratios were notably different than unadjusted estimates, these changes were likely due to over-fitting models with a small sample size. Therefore adjusted estimates are not presented in this paper, but can be found in the supplementary files.

### Perceived facilitators and barriers

Interviews with health workers and observations by the study teams showed varied patterns of PMTCT services with substantial variance in facilitators and barriers to care. When data from interviews and observations were analysed by performance group, three of the top five facilitators cited at high-performing sites also appeared in the top five barriers cited at low-performing sites. These included adequate space (high/facilitator – 8 sites; low/barrier – 8 sites); ventilation (high/facilitator – 8 sites; low/barrier – 6 sites) and the availability of trained staff (high/facilitator – 7 sites; low/barrier – 4 sites). Thus, the presence or absence of these three factors was perceived to have a high impact on overall PMTCT performance. Other perceived factors influencing patient retention included the availability of medicines, tests, supplies, and equipment and conditions for confidentiality.

Although patient flow generally followed MSHP guidelines for PMTCT at all sites, the level of the integration of the HIV services into ANC and timing of enrolment in care varied substantially among sites. Fourteen sites conducted HIV testing in the ANC room (as in Figure 1), while others referred women to another room in a different part of the health facility for the rapid test. Some health workers preferred a separate room for testing for perceived greater confidentiality and efficiency. Other respondents expressed concerns that a separate testing room added to the total time a woman spent receiving overall ANC and PMTCT services.

Patient flow and wait times were frequently cited as both perceived facilitators and barriers to care. Long wait times were often reported in conjunction with insufficient staffing levels and/or appropriate training. For example, a doctor at one urban site noted: “There is only one antenatal consultation room at this health facility. This means that there is only one health provider who delivers ANC services each day. When there are busy days, wait times can be very long.” Inversely, some respondents noted low wait times as a result of the availability and training of staff. At one rural site, a key informant noted the impact of having a “PEPFAR-supported laboratory with qualified personnel” on reducing wait times and consequently LTFU during the CD4 testing stage.

## Discussion

This nationally representative, random sample of antenatal clinics providing PMTCT services in Côte d'Ivoire demonstrated significant losses and delays throughout the PMTCT cascade, from the identification and delivery of appropriate prophylaxis to infant testing and likely adherence to lifelong treatment. These findings are consistent with previous studies that have demonstrated LTFU in the PMTCT cascade in African countries at similar levels [17].

The multiplicity of registries and other data sources likely contributed to inconsistent record keeping and reported data. These inconsistencies – especially with respect to the lower proportion of women tested for HIV reported in the carnets than in registries – raised concerns about the validity of data from standard sources. In the long term, the authors support the improvement of indicator sensitivity and reducing redundancies of data collection. However, in the short term, the use of data from multiple sources can help identify areas for improvement within the existing health information system and note possible undocumented loss – such as the 17% of women lacking documentation of HIV testing in their carnet.

Despite WHO guidelines requiring CD4 testing to determine appropriate prophylaxis under Option A, this study showed that many women received ARVs without undergoing CD4 testing. This suggests that the CD4 test requirement was not the principal barrier to prophylaxis delivery, and that its elimination under Option B/B+ will need to be supplemented with additional strategies to encourage the uptake of ARVs. Further research on specific systems-level interventions will be required following the transition to the option B/B+ regimens to reduce LTFU at this stage in the cascade.

Another finding with implications for Option B/B+ was the low level of adherence to lifelong treatment approximated by using patient chart data. It is likely that actual cART adherence rates may have been higher than the proxy indicator used in this study suggested, as interviewers at several sites noted that many patient visits or transfers were not recorded in patient charts. Patient tracking systems should be highlighted under Option B/B+ to better understand retention following initiation of cART. Such systems would serve to both improve data quality for decision-making, and identify patients at high risk for LTFU [18].

The wide variability of PMTCT scores among sampled clinics suggests that major improvements in PMTCT service effectiveness are possible with attention to the factors noted in this study, such as the training and availability of key human resources and delays in processes of care. Interviews with frontline health workers reinforced these findings and suggested that human resource factors do more to facilitate service delivery, while infrastructural and material deficiencies serve as the most tangible barriers to care. These findings are consistent with previous studies of PMTCT programmes in Sub-Saharan Africa [19,20]. At the policy level, staffing and training should be prioritised. At the facility level, implementable systems-based interventions are needed, such as improvement of physical conditions (space, ventilation, cleanliness) and ensuring the systematic enrolment, documentation and tracking of patients in care.

### Limitations

Although this mixed-method assessment was done with a robust national sample of 30 PMTCT sites, the assessment was limited by several factors. Registry analyses were cross-sectional, limiting inferences of causality. When adjusted models were used to attempt to control for confounding in the analysis of correlates of high and low performance, the resulting estimates were imprecise. This was likely due to over-fitting models in a small sample. The continued presence of confounders means that our results must be interpreted cautiously, but this caution should be weighed against the strong scientific plausibility of the likelihood that staffing and training levels influence PMTCT outcomes.

Estimates of LTFU assumed that patients continue to seek PMTCT services at the same health facility. If health facilities had referred out for certain services due to lack of equipment or periodic stock shortages, those patients would not have been accounted for in retention rates. Additionally, key informants were health care facility staff; no patient perspectives were elicited regarding barriers and facilitators to care.

As mentioned above, poor data quality and availability served as both a limitation and opportunity for analysis. Missing data, such as proof of testing in carnets, may have represented non-reporting rather than the failure to provide care. Similarly, the patient chart sample, which likely overrepresented HIV-positive women on lifelong cART, may include non-reporting (missing clinical visits; missing charts) or the failure to provide care. This impacted the extent to which carnet and chart-based inferences could be extended to all women in PMTCT care.

Last, though the use of a composite PMTCT score to identify high- and low-performing sites helped to inform areas for intervention, using different measures of retention in the calculation of the PMTCT score would have changed the distribution of sites into low- and high-performance categories. Various combinations of retention indicators and scoring methodologies were tested with the final methodology based on advice from MSHP officials.

## Conclusions

This study provided a rapid method to assess national PMTCT programme performance at the health facility level and identify factors associated with high and low performance. In less than two weeks, the study team was able to capture both quantitative and qualitative data from multiple data sources that demonstrated strengths and weaknesses of the PMTCT programme not typically apparent from routine reporting. Despite inconsistencies in both the quality and availability of data across the sample, the combination of multiple existing data sources helped to identify key characteristics of high- and low-performing sites, which should inform interventions to reduce LTFU in the PMTCT cascade.

This study suggests that strategies to improve patient retention and decrease MTCT under Option B/B+ should combine efforts to increase the availability and training of key frontline health workers, improve physical conditions at ANC clinics to enhance patient care and confidentiality and simplify and strengthen data collection systems. A systems focus on patient follow-up with adequate charting and communication with patients is a critical starting point for improving patient retention.

## Supplementary Material

Results from a rapid national assessment of services for the prevention of mother-to-child transmission of HIV in Côte d'Ivoire
