# Supplementary material for: Results from a rapid national assessment of services for the prevention of mother-to-child transmission of HIV in Côte d'Ivoire
Source: J Int AIDS Soc. 2016 Jul 20;19(5Suppl 4):20838. doi: 10.7448/IAS.19.5.20838 (PMC4956736; doi:10.7448/IAS.19.5.20838)
Supplement: Results from a rapid national assessment of services for the prevention of mother-to-child transmission of HIV in Côte d'Ivoire [file JIAS-19-20838-s001.pdf]

Supplementary Table 1. Logistic regression analyses evaluating site characteristics as correlates of high or low performance in health facilities providing PMTCT services in Côte d'Ivoire

Site characteristic	Low: High performing sites in muti-linear model	Univariate model Odds ratio [95% CI]	Multivariate model Odds ratio [95% CI]
**Processes of care**			
Days elapsed between HIV test given and CD4 results received (recorded in patient charts)[1]	--	--	--
Days elapsed between ANC1 visit and CD4 results (reported by staff) [2]	10:10	2.19 [1.27, 3.78] p = 0.005	2.49 [1.41, 4.40] p= 0.002
**Workforce distribution**			
Number of full-time physicians per 100 ANC1 patients[3,4]	7:8	0.62 [0.42, 0.94] p = 0.023	0.37 [0.15, 0.94] p = 0.036
Number of laboratory technicians per 100 ANC1 patients[3,4]	7:8	0.51 [0.21, 1.27] p = 0.150	0.28 [0.08, 0.99] p= 0.048
Number of nurses per 100 ANC1 patients[3,4]	7:8	0.83 [0.71, 0.96] p = 0.014	0.73 [0.48, 1.11] p = 0.142
**Workforce training**			
Number of staff trained in PMTCT per 100 ANC1 patients[3,5]	10:10	0.56 [0.34, 0.92] p = 0.022	0.09 [0.01, 0.91] p = 0.041
Number of staff who conduct follow up per 100 ANC1 patients[3,5]	10:10	0.46 [0.20, 1.06] p = 0.069	0.30 [0.11, 0.81] p = 0.018

[1]Estimate for this variable could not be adjusted. Due to the small number of patient charts reviewed this data was pooled at group (ie: low performing group or high performance group level), rather than being pooled to the individual clinic level.

[2]Adjusted for Urban/Rural location and average ANC1 visits per month. Catchment population was not included as the model would not converge with variable included.

[3]All independent workforce variables were pre-adjusted for average ANC1 visits per month, so this variable was excluded from the model for workforce distribution and workforce training characteristics.

[4]Workforce distribution characteristics were adjusted for Urban/Rural location and catchment population size. Five sites were missing data on the size of their catchment population, reducing the N of these models.

[5]Workforce training characteristics were adjusted for Urban/Rural location, but not adjusted for catchment population size due to an inability to get the models to converge.
